# Acupuncture and moxibustion for lateral elbow pain: a systematic review of randomized controlled trials

**DOI:** 10.1186/1472-6882-14-136

**Published:** 2014-04-12

**Authors:** Marcus Gadau, Wing-Fai Yeung, Hua Liu, Chris Zaslawski, Yuan-Sheng Tan, Fu-Chun Wang, Sergio Bangrazi, Ka-Fai Chung, Zhao-Xiang Bian, Shi-Ping Zhang

**Affiliations:** 1 School of Chinese Medicine, Hong Kong Baptist University, Hong Kong, SAR, China; 2 Department of Psychiatry, University of Hong Kong, Hong Kong, SAR, China; 3 Department of Neurology, the Second Clinical Medical College of North Sichuan Medical College, Nanchong, China; 4 College of Traditional Chinese Medicine, University of Technology Sydney, Sydney, Australia; 5 World Federation of Acupuncture and Moxibustion Societies, Beijing, China; 6 Changchun University of Traditional Chinese Medicine, Jilin, China; 7 Instituto Paracelso, Roma, Italy; 8 From the Tennis Elbow Acupuncture International Study-China, Hong Kong, Australia and Italy (TEA-IS-CHAI) group

**Keywords:** Epicondylitis, Tennis elbow, Randomized Controlled Trials (RCTs), STRICTA, PRISMA, Cochrane risk of bias tool, Chinese literature

## Abstract

**Background:**

Acupuncture and moxibustion have widely been used to treat lateral elbow pain (LEP). A comprehensive systematic review of randomized controlled trials (RCTs) including both English and Chinese databases was conducted to assess the efficacy of acupuncture and moxibustion in the treatment of LEP.

**Methods:**

Revised STRICTA (2010) criteria were used to appraise the acupuncture procedures, the Cochrane risk of bias tool was used to assess the methodological quality of the studies. A total of 19 RCTs that compared acupuncture and/or moxibustion with sham acupuncture, another form of acupuncture, or conventional treatment were included.

**Results:**

All studies had at least one domain rated as high risk or uncertain risk of bias in the Cochrane risk of bias tool. Results from three RCTs of moderate quality showed that acupuncture was more effective than sham acupuncture. Results from 10 RCTs of mostly low quality showed that acupuncture or moxibustion was superior or equal to conventional treatment, such as local anesthetic injection, local steroid injection, non-steroidal anti- inflammatory drugs, or ultrasound. There were six low quality RCTs that compared acupuncture and moxibustion combined with manual acupuncture alone, and all showed that acupuncture and moxibustion combined was superior to manual acupuncture alone.

**Conclusion:**

Moderate quality studies suggest that acupuncture is more effective than sham acupuncture. Interpretations of findings regarding acupuncture vs. conventional treatment, and acupuncture and moxibustion combined vs. manual acupuncture alone are limited by the methodological qualities of these studies. Future studies with improved methodological design are warranted to confirm the efficacy of acupuncture and moxibustion for LEP.

## Background

Lateral elbow pain (LEP), commonly known as tennis elbow, is a common disorder with a prevalence of at least 1-3% [1]. It is a significant health burden because it affects work productivity and the quality of life of LEP sufferers. Currently there is no ideal treatment for LEP. The most common treatments for LEP are steroid injections, non-steroidal anti-inflammatory drugs (NSAIDs) or a regime of physiotherapy with various modalities [2]. Steroid injections have a short-term (two to six weeks) effect in improving symptoms [3], whereas NSAIDs have a smaller effect than steroid injections [4]. Evidence is lacking for the efficacy of physiotherapy [5]. Furthermore, there is no evidence regarding the efficacy in the long term use of current conservative treatment options, and the potential side effects such as skin atrophy and depigmentation [6] limit the use of steroid injections. The need for a safe and effective treatment for LEP is therefore paramount.

Acupuncture is a popular form of complementary and alternative medicine for treating pain and dysfunction associated with musculoskeletal conditions [7], including LEP. In traditional Chinese medicine (TCM), acupuncture and moxibustion are two inseparable therapeutic methods; the former stimulates the acupoint with a needle whereas the latter with heat generated by burning of moxa (Artemisia Vulgaris). Acupuncture has been popularly used all over the world, but it is still not recognized as a standard treatment for LEP because the evidence supporting its efficacy is still limited. In the most recent review on the topic, Buchbinder et al. [8] evaluated five randomized controlled trials (RCTs) comparing acupuncture with sham acupuncture. The authors concluded that needle acupuncture may be more effective than sham acupuncture in relieving pain after one treatment as well as after ten acupuncture sessions at two weeks, but there is no difference between needle acupuncture and sham acupuncture at the 3-or 12-month follow-up. They also found that needle acupuncture may be more effective at improving functional impairment at 2-week follow-up compared with sham acupuncture, and electro-acupuncture may be more effective than manual acupuncture in reducing pain. Nevertheless, they concluded the acupuncture intervention as "unknown effectiveness" in their report, due to the fact that the analyzed studies were of very small sample size and had flaws in the study design, including uncertain allocation concealment, substantial loss to follow up and lack of assessments for potential adverse effects.

Although a number of systematic reviews have been performed on acupuncture for LEP [4,8-10], these reviews did not include articles published in Chinese. Moreover, moxibustion, which also acts by stimulating the acupoints and is commonly used concurrently with acupuncture for LEP, was not included in previous reviews. Given the fact that many studies of acupuncture and moxibustion for LEP have been published in non-Western scientific literature and have not been reviewed, the literatures identified by previous reviews may not be comprehensive enough to cover all the current evidence of acupuncture and moxibustion for LEP. In view of this, we conducted a comprehensive systematic review on randomized controlled trials of acupuncture and moxibustion for LEP that were published in both Chinese and Western language literatures. This review aimed to find out if acupuncture or moxibustion alone was more effective than sham acupuncture or other conventional treatments in the treatment of LEP. We also wanted to know if acupuncture and moxibustion combined was more effective than acupuncture or moxibustion alone. In addition, this review evaluated reporting of acupuncture and/or moxibustion treatment of the included studies using the revised STRICTA criteria [11].

## Methods

### Search strategies

A comprehensive search was performed in the following databases from their inception to June 2013: Cochrane Neuromuscular Disease Group Trials Register for randomized trials, the Cochrane Central Register of Controlled Trials (CENTRAL), MEDLINE, EMBASE, Latin American and Caribbean Health Sciences (LILACS), Allied and Complementary Medicine (AMED), Index to Chinese Periodicals of Hong Kong (HKInChiP), Chinese Biomedical Literature Database (CBM) and China National Knowledge Infrastructure (CNKI). Search was also made in ClinicalTrials.gov, ProQuest Digital Dissertations (PQDD), BIOSIS Previews, Chinese Clinical Trial Register (ChiCTR), and Electronic Theses and Dissertations System of Taiwan for “gray literature”, such as unpublished studies, dissertations and conference reports. The following terms were used in our search strategies: (moxibustion or acupuncture or electro-acupuncture or needle) and (lateral epicondylitis or, lateral epicondylalgia, or tennis elbow, or lateral epicondyle, or external humeral epicondylitis, or brachioradial bursitis, or lateral elbow pain, or lateral elbow). Equivalent Chinese terms were used in searching the Chinese language databases. We imposed no language restrictions. We followed the Preferred Reporting Items for Systematic Reviews and Meta-Analyses (PRISMA) in reporting this systematic review [12].

### Study selection

Two authors (MG and WFY) searched the databases and assessed potentially relevant articles against the inclusion criteria independently. Any disagreement regarding the eligibility of a study was resolved by discussion.

### Types of studies and subjects

We included only randomized controlled trials studying subjects with a primarily diagnosis of lateral epicondylitis or lateral elbow pain, in which acupuncture, moxibustion, or acupuncture and moxibustion combined was used for treatment.

### Types of interventions

#### Acupuncture

Acupuncture is defined as needle acupuncture, including electro-acupuncture and auricular acupuncture that employed needle penetration. Other variants of acupuncture, such as acupressure, acupoint injection, laser acupuncture, auricular acupressure, and transcutaneous electrical nerve stimulation (TENS) were excluded. Treatments that used acupotomy (small needle-scalpel therapy) were also excluded.

#### Moxibustion

Moxibustion is defined as burning of moxa either directly or indirectly over acupoints. In direct moxibustion, the moxa is placed directly over the skin. In indirect moxibustion, the burning moxa is positioned over the acupoint, either held by an apparatus, or placed over a piece of herbal material, such as ginger or biscuit made from medicinal herbs.

#### Acupuncture and moxibustion combined (AMC)

We also included studies of combining acupuncture and moxibustion treatment (acupuncture and moxibustion combined, AMC) which is usually done by placing a moxa block on the handle of the acupuncture needle or placing a moxa-cone on top of a thin piece of ginger-slice. Studies using acupuncture or moxibustion in combination with other treatments, such as medication, massage, cupping, physiotherapy, traditional Chinese herbs, or injection were not included.

### Types of control interventions

For control interventions, we included studies that used other standard therapies, such as injection of Western drugs, physiotherapy, oral Western medication, sham acupuncture, or no treatment. We also included studies that compared AMC with either acupuncture or moxibustion alone. However, we did not include studies that compared the same intervention with different combinations of acupoints, as acupoint specificity was not the focus of this review.

### Data extraction

One author (MG) extracted the data and the other (WFY) checked the extracted data. For each study, the following variables were extracted: study design, patients’ characteristics including gender, age, duration of illness, treatment regime, control intervention, and outcome measures and adverse events reported.

Primary outcomes extracted included measures on pain and function. Secondary outcomes extracted included measures on quality of life. The details of acupuncture procedure were extracted according to the revised STRICTA [11], which covered acupuncture rationale, needling details, treatment regime, other components of treatment, practitioner background, and control intervention.

### Methodological quality assessment

The methodological quality of identified studies was assessed by two authors (MG and WFY) independently using the Cochrane risk of bias tool [13]. The Cochrane risk of bias assessment has 6 domains: random sequence generation, allocation concealment, blinding of participants and outcome assessors, complete collection and reporting of outcome data, free of selective outcome reporting, and adequate attention to other sources of bias. Each domain was rated as “low” (low risk of bias), ‘high’ (high risk of bias), or ‘unclear’ (uncertain risk of bias). Given the difficulties in blinding the acupuncturist, we only assessed the blinding of participants and outcome assessors.

According to a recent report by Wu et al. [14], a large proportion of RCTs in Chinese language reports are not truly randomized. Therefore a telephone inquiry to the first authors, and, if they were not available, to the second authors, was conducted to find out how many of them truly met acceptable standards for allocating participants to study groups.

### Statistical analysis

We used Review Manager Software 5.1 for statistical analysis. Relative risk (RR) with a 95% confidence interval (CI) was used for dichotomous outcomes. Mean difference (MD) with a 95% CI was used for continuous outcomes. Publication bias would be assessed by drawing a funnel plot if there were ten studies or more included in the meta-analysis. Meta-analysis would only be performed, if studies had no domain rated as having high risk of bias by the Cochrane risk of bias assessment and had sufficient similarities in clinical characteristics [13].

## Results

### Selection of studies

**Figure 1:**
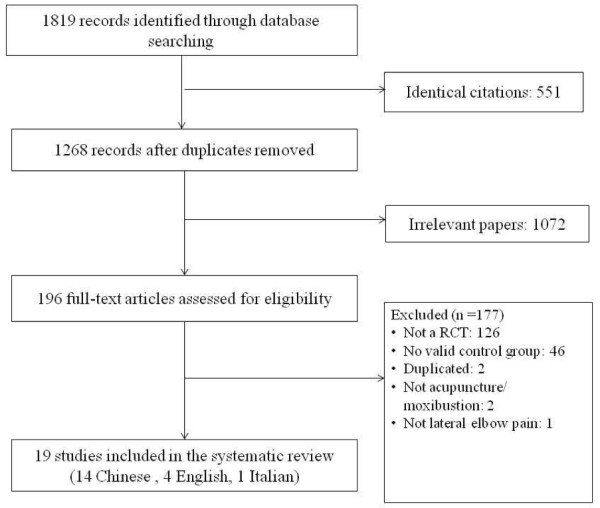
Flow chart of study selection.

The search identified 580 English and 1239 Chinese potentially relevant citations for review. After removal of duplicates, 232 English and 1036 Chinese citations were left. There were 206 English and 866 Chinese papers excluded for reasons of irrelevance (Figure 1). One hundred ninety-six full-text articles were retrieved for further assessment. Two studies were duplicated publications [15,16]. Finally, 19 studies (14 Chinese, four English, one Italian) met the inclusion criteria and were included in this review.

### Description of included studies

All 19 included studies were full length journal reports. Of the 19 studies, 14 were published in Chinese and were conducted in China; four were published in English, three of which were conducted in Germany [15-17] and one was conducted in Canada [18]. The remaining study was conducted in Italy and published in Italian [19]. Together these studies involved a total of 1190 subjects, with 688 being in the treatment arm and 502 in the control arm. All included trials used a two-armed, parallel group design, except for the Shen et al. study [20], which used a three-armed, parallel group design.

The sample size of the included studies ranged from 16 to 120 subjects. All subjects were out-patients, with an age range from 17 to 74 years in the treatment arm and 20 to 76 years in the control arm. The terminology used for LEP varied between studies. Twelve studies [18-29] used the term lateral epicondylitis, whereas the term tennis elbow was used by four studies [30-33] to describe the condition. One study used both terms in its title, but in the text consistently used the term lateral epicondylitis [17]. One study used the term chronic epicondylitis [15] and the remaining study used the term chronic elbow pain [16]. We decided to use the term LEP, rather than epicodylitis as research has shown that the pathophysiology of tennis elbow is a breakdown of the tendon (tendinosis) rather than inflammation [34]. In this review the term LEP refers to pain at the lateral side of the elbow region, especially to pain which originates from the lateral epicondyle. We refrained from using the term “chronic” due to the fact that many studies included subjects with an elbow pain duration too recent to be classified as chronic. The duration of the LEP described in the included studies varied from seven days to five years. Follow-up periods varied from one day up to one year after the last treatment. Table 1 presents the characteristics of all included studies.

**Table 1:** Study design of all included studies

**1st Author (year)**	**Design, Follow-up**	**Intervention, treatment duration**		**Details of intervention**	
		**Treatment group**	**Control group**	**Treatment group**	**Control group**
Chen (2010) [30]	2 parallel arms, follow-up duration not reported	Acupuncture, 1 treatment every 3 days for 15 days	Triamcinolone acetonide injection, 1 treatment every 5 days for 15 days	Local tender points, superficial, strong manual manipulation, needles retained for 1 min.	Triamcinolone acetonide 40 mg, 2% lidocaine 2 ml injected at area of pain
Davidson (2001) [18]	2 parallel arms, follow-up duration not reported	Acupuncture, 2–3 treatments per week for 4 weeks	Ultrasound, 2–3 treatments per week for 4 weeks	LI 4, SJ 5, LI 10, LI 11, LI 12, manual manipulation to obtain and maintain De-qi, needles retained for 20 min.	Pulsated ultrasound for 10 min. over area of lateral epicondyle
Fink (2002) [15]	2 parallel arms, 2 months	Acupuncture, 2 treatments per week for 5 weeks	Sham-acupuncture, 2 treatments per week for 5 weeks	1 local tender point, LI 10, LI 11, LU 5, LI 4 and SJ 5, manual manipulation to obtain De-qi, needles retained for 25 min.	Same as treatment group, but needles placed at least 5 cm away from real acupoint, area clear of tender points
Grua (1999) [19]	2 parallel arms, follow-up duration not reported	Acupuncture, 1–2 treatments per week for approx. 5 weeks (total of 10 treatments)	Ultrasound, massage, 1 treatment per day for 12 days	LI 4, LI 10, LI 11, LI 12, LI 15, PC 5, PC 7, GB 20, GB 21, GB 34, ST 37, ST 38, manual manipulation needles retained for 20 min.	Pulsated ultrasound for 5 min., massage for 5 min., both at area of lateral epicondyle
Irnich (2003) [17]	2 parallel arms, 2 weeks	Acupuncture, 3 treatments for 10 days	Sham-acupuncture, 3 treatments for 10 days	LI 4, LI 10, SI 3, SJ 5, GB 34, intermittent manual manipulation to obtain and maintain De-qi, needles retained for 25 min.	Same as treatment group, but needles placed 1 cun away from real acupoint
Jin (2005) [33]	2 parallel arms, 1 month	Single scarring ginger- moxibustion	Prednisolone compound injection, 1 treatment per week for 2 weeks	Local tender points, ginger- moxibustion, 7 cones per acupoint	2% lidocaine 1 ml, prednisolone 1 ml, Vitamin B1 50 mg, Vitamin B12 250 μg injected at area of pain
Li (1998) [28]	2 parallel arms, follow-up duration not reported	AMC, 1 treatment every 1 or 2 days for 2 months	Acupuncture, 1 treatment every 2 days for 2 months	Local tender points, LI 4, LI 10, LI 11, AMC, manual stimulation to obtain and maintain De-qi, needles retained for 15–20 min., moxibustion with moxa-stick until local area reddened	Same as treatment group, but only manual stimulation to obtain and maintain De-qi
Li (2007) [22]	2 parallel arms, follow-up duration not reported	Ginger-moxibustion, 1 treatment every 2 days for 14 days	Prednisolone injection, 1 treatment every 5–7 days for 14–21 days	SJ 10, LI 11, manual stimulation to obtain De-qi, needles not retained, ginger-moxibustion 5–7 cones per acupoint	2% lidocaine 2 ml, prednisolone 25 mg injected at area of pain
Liu (2008) [23]	2 parallel arms, follow-up duration not reported	AMC, 1 treatment every 2 days for 28 days	Acupuncture, 1 treatment every 2 days for 28 days	Local tender points, LI 4, LI 10, LI 11, SJ 5, AMC, manual stimulation to obtain and maintain De-qi, needle retained for 20–30 min., indirect moxibustion, 3–5 cones per acupoint	Same as treatment group, but only manual stimulation to obtain and maintain De-qi
Lin (2011) [21]	2 parallel arms, 1 month	Acupuncture, 1 treatment every 2 days for 20 days	Prednisolone injection, 1 treatment every 10 days for 20 days	LI 11, 1 most tender point on lateral aspect of elbow, 3 points 0.5-1 cun distal to the most tender point, manual manipulation to obtain and maintain De-qi, needles retained for 30 min.	Prednisolone 5 ml, 2% procaine 1.5 ml injected at area of pain
Molsberger (1994) [16]	2 parallel arms, 3 days	Acupuncture, 1 treatment only	Sham-acupuncture, 1 treatment only	Ipsilateral GB 34, manual manipulation to obtain and maintain De-qi, needles retained for 5 min.	BL13 non-needle sham acupuncture, stimulation with a pencil-like probe at beginning and after 5 min.
Shen (1999) [20]	3 parallel arms, follow-up duration not reported	Electro-acupuncture, 1 treatment per day for 10 days	Control 1: AMC, 1 treatment per day for 10 days	Local tender points, manual manipulation to obtain De-qi followed by electric stimulation for 30 min., heat lamp for 30 min.	Control 1: Same as treatment group but AMC, needle retainment for 30 min., indirect moxibustion (moxa stick)
			Control 2: Prednisolone, 1 treatment per week for 3 weeks		Control 2: Prednisolone 20 mg injected at area of pain
Wang (2007) [24]	2 parallel arms, follow-up duration not reported	AMC, 1 treatment per day for 10 days	Acupuncture, 1 treatment per day for 10 days	LI 10, LI 11, SJ 10, PC 6, REN 12, ST 36, SP 6, points chosen based on TCM pattern diagnosis, AMC, De-qi obtained, duration of needle retainment not reported, indirect moxibustion 3 cones per acupoint	Same as treatment group, but only manual stimulation to obtain De-qi, no additional stimulation during treatment, duration of needle retainment not reported
Wang (2008) [25]	2 parallel arms, follow-up duration not reported	AMC, 1 treatment every 3 days for 30 days	Acupuncture, 1 treatment every 3 days for 30 days	5 local tender points, LI 4, AMC, even manual stimulation technique to obtain and maintain De-qi, needles retained for 30 min., indirect moxibustion, 2–3 cones per acupoint	LI 4, LI 10, LI11, LI 12, manual stimulation every 10 min., needles retained for 30 min.
Wu (2003) [26]	2 parallel arms, follow-up duration not reported	AMC, 1 treatment every 2 days for 14 days	Acupuncture, 1 treatment every 2 days for 14 days	Local tender points, LI 4, LI 10, LI 11, SJ 5, manual manipulation for 1 min. to obtain and maintain De-qi, needles retained for 30 min., indirect moxibustion 3–5 cones per acupoint	1 local tender point was selected, De-qi obtained, needles retained for 30 min.
Xu (2010) [29]	2 parallel arms, follow-up duration not reported	Single scarring ginger-moxibustion	Prednisolone injection, 1 treatment every 5 days for 15 days	Local tender points, ginger- moxibustion, duration not reported	Prednisolone 25 mg, 2% procaine 2 ml injected at area of pain
Zha (2004) [31]	2 parallel arms, follow-up duration not reported	Acupuncture, 1 treatment every 2 days for 14 days	Hydrocortisone acetate injection, 1 treatment per week for two weeks	Local tender points, manual manipulation, duration of needle retainment not reported	2% lidocaine 5 ml, hydrocortisone-acetate 125 mg (1 ml) injected at area of pain.
Zhang (2007) [32]	2 parallel arms, follow-up duration not reported	Acupuncture, 1 treatment every 2 days for 20 days	Meloxicam tablets oral intake, 1 treatment every 2 days for 20 days	1 local tender point and 2 points at 2 cm apart from the tender point, manual manipulation to obtain and maintain De-qi, duration of needle retainment not reported	Meloxicam tablets 7.5 mg/ day
Zhao (2003) [27]	2 parallel arms, follow-up duration not reported	AMC, 1 treatment per day for 10 days	Acupuncture, 1 treatment per day for 10 days	Local tender points, manual manipulation to obtain and maintain De-qi, duration of needle retainment not reported, indirect moxibustion (moxa stick) until local area reddened	Acupuncture at local tender points, manual stimulation, duration of needle retainment not reported

AMC: acupuncture and moxibustion combined.

Acupuncture was compared with sham acupuncture in three studies (Table 2). One study [16] used non-invasive sham acupuncture at BL 13 on the back, in which subjects were stimulated with a pencil like probe and were shown an acupuncture needle. The other two studies [15,17] inserted real acupuncture needles a few centimeters away from traditional acupoints, their interconnecting lines (meridians) and painful pressure points.

**Table 2:** Outcomes of randomized controlled trials comparing acupuncture and sham acupuncture

**Studies**	**Number of subjects, intervention**		**Outcome measurement**	**Outcomes**		**Treatment effect**
	**Treatment group**	**Control group**		**Treatment group**	**Control group**	
Fink (2002) [15]	Acupuncture	Sham-acupuncture	Strength test (peak muscle force):			MD (95% CI)
	N = 14	N = 15	mean ± SD	At baseline: 90.5 ± 40.40	At baseline: 77.7± 36.40	12.80 (−15.26 to 40.86), P = 0.37
				At 2 weeks FU: 128.2 ± 41.64	At 2 weeks FU: 92.75 ± 34.78	35.45 (7.42 to 63.48), P = 0.01*
				At 2 months FU: 142.9 ± 41.56	At 2 months FU: 114.2 ± 46.08	28.70 (−3.20 to 60.60), P = 0.08
		
			Pain (VAS): mean ± SD	At baseline: 16.46 ± 3.10	At baseline: 17.17 ± 3.76	−1.24 (−3.74 to 1.26), P = 0.33
				At 2 weeks FU: 8.03 ± 4.60	At 2 weeks FU: 12.28 ± 4.14	−4.25 (−7.44 to −1.06), P = 0.009*
				At 2 months FU: 6.01 ± 5.09	At 2 months FU: 8.73 ± 5.03	−2.72 (−6.41 to 0.97), P = 0.15
			
			DASH scores: mean ± SD	At baseline: 38.08 ± 13.66	At baseline: 33.72 ± 13.05	4.36 (−5.38 to 14.10), P = 0.38
				At 2 weeks FU: 14.38 ± 9.35	At 2 weeks FU: 25.18 ± 13.63	−10.80 (−19.26 to −2.34), P = 0.01*
				At 2 months FU: 11.14 ± 13.10	At 2 months FU: 18.85 ± 13.75	−7.71 (−17.48 to 2.06), P = 0.12
Irnich (2003) [17]	Acupuncture	Sham-acupuncture	Pressure pain threshold (kg/cm^2^):			
	N = 25	N = 25	mean ± SD	At baseline: 3.15 ± 0.69	At baseline: 2.68 ± 0.65	0.47 (0.10 to 0.84), P = 0.01*
				At first treatment: 0.32 ± 0.31	At first treatment: 0.16 ± 0.176	0.16 (0.02 to 0.30), P = 0.02*
				At last treatment: 0.93 ± 0.49	At last treatment: 0.63 ± 0.42	0.30 (0.05 to 0.55), P = 0.02*
				At 2 weeks FU: 1.30 ± 0.60	At 2 weeks FU: 0.66 ± 0.49	0.64 (0.34 to 0.94), P < 0.0001*
		
			Pain-free grip strength:	At baseline: 64.7 ± 34.00	At baseline: 53.7 ± 17.70	11.00 (−4.03 to 26.03), P = 0.15
			mean ± SD	At first treatment: 7.12 ± 8.13	At first treatment: 2.47 ± 3.14	4.65 (1.23 to 8.07), P = 0.008*
				At last treatment: 21.54 ± 14.35	At last treatment: 8.53 ± 9.05	13.01 (6.36 to 19.66), P = 0.0001*
				At 2 weeks FU: 27.95 ± 15.66	At 2 weeks FU: 7.4 ± 7.90	20.55 (13.67 to 27.43), P < 0.00001*
		
			Impairment caused by pain (NRS):	At baseline: 8.19	At baseline: 7.72	Calculation of MD not possible
				At first treatment: −1.57	At first treatment: −0.80	
				At last treatment: −4.31	At last treatment: −2.04*	
				At 2 weeks FU: −4.77 (59% mean decrease in impairment caused by pain)	At 2 weeks FU: −1.88* (24% mean decrease in impairment caused by pain)	
			
Molsberger (1994) [16]	Acupuncture	Sham-acupuncture	11-point box pain scale (NRS):	Immediately after treatment:	Immediately after treatment:	MD (95% CI)
	N = 24	N = 24		55.8% (2.95) mean pain reduction,	15.0% (2.77) mean pain reduction,	40.80 (39.18 to 42.42), P < 0.001*
				19/24 subjects reported pain relief of ≥ 50% (NRS ≥ 5)	6/24 subjects reported pain relief of ≥ 50% (NRS ≥ 5)	RR (95% CI) 3.17 (1.53 to 6.52), P < 0.002*

*: P < 0.05 statistically significant difference between two groups.

DASH score, Disability of the Arm, Shoulder and Hand score; FU, follow-up; NRS, Numeric Rating Scale; SD, Standard Deviation; MD, Mean Difference; RR, Relative Risk; VAS, Visual Analogue Rating Scale.

Seven trials compared acupuncture with conventional therapies, of which four studies [20,21,30,31] used local injection of steroid and/or local anesthetics as the control intervention. One [18] used pulsated ultrasound, one used pulsated ultrasound in combination with massage [19], and the remaining one [32] used meloxicam tablets as the control intervention (Table 3).

**Table 3:** Outcomes of randomized controlled trials comparing acupuncture and conventional therapy

**Studies**	**Intervention and number of subjects**		**Outcome measurement**	Outcomes**		**Treatment effect**
	**Treatment group**	**Control group**		**Treatment group**	**Control group**	
Chen (2010) [30]	Acupuncture N = 33	Triamcinolone acetonide injection N = 33	Pain relief and grip strength: cured rate based on subjective report	30/33 Cured Cured rate: 92.4%	14/33 Cured Cured rate: 42.4%	RR (95% CI) 2.14 (1.42 to 3.23) P = 0.0003*
Davidson (2001) [18]	Acupuncture N = 8	Pulsated ultrasound N = 8	Pain-free grip strength: mean ± SD	At first treatment: 10.25 ± 5.84	At first treatment: 6.08 ± 4.19	MD (95% CI) 4.17 (−0.81 to 9.15), P = 0.10.
				At last treatment: 14.09 ± 9.53	At last treatment: 11.96 ± 12.28	2.13 (−8.64 to 12.90), P = 0.70
			Pain Score (VAS): mean ± SD	At first treatment: 39.63 ± 29.51	At first treatment: 46.50 ± 26.91	−6.81 (−34.48 to 20.86), P = 0.63
				At last treatment: 13.63 ± 13.79	At last treatment: 32.69 ± 29.21	−19.06 (−41.44 to 3.32), P = 0.10
			DASH score: mean ± SD	At first treatment: 36.35 ± 25.54	At first treatment: 38.02 ± 15.24	−1.67 (−22.28 to 18.94), P = 0.87
				At last treatment: 23.75 ± 17.73	At last treatment: 33.23 ± 24.06	−9.48 (−30.19 to 11.23), P = 0.37
				At 4 week FU: 23.75 ± 18.41	At 4 week FU: 22.40 ± 18.73	1.35 (−16.85 to 19.55), P = 0.88
Grua (1999) [19]	Acupuncture N = 20	Pulsated ultrasound, massage N = 20	Maigne functional recovery test: mean ± SD	At first visit: 15.0 ± 2.36	At first visit: 15.0 ± 2.70	MD (95% CI) 0.00 (−1.57 to 1.57), P = 1.00
					At last treatment: 5.80 ± 3.37	At last treatment: 9.80 ± 3.65	−4.00 (−6.18 to −1.82), P = 0.0003*
				At 6 months FU: 5.20 ± 3.64	At 6 months FU: 10.0 ± 3.45	−4.80 (−7.00 to −2.60, P < 0.0001*	
			Pain Score(VAS): mean ± SD	At first visit: 7.05 ± 1.47	At first visit: 7.05 ± 1.39	0.00 (−0.89 to 0.89), P = 1.00	
				At last treatment: 2.85 ± 1.81	At last treatment: 4.49 ± 1.64	−1.64 (−2.71 to −0.57), P = 0.003*	
				At 6 months FU: 2.05 ± 1.39	At 6 months FU: 4.90 ± 1.45	−2.85 (−3.73 to −1.97), P < 0.00001*	
Lin (2011) [21]	Acupuncture N = 36	Prednisolone injection N = 36	Pain relief: VAS, cured rate based on subjective report	20/36 Cured Cured rate: 55.5%	13/36 Cured Cured rate: 36.1%	RR (95% CI) 1.54 (0.91 to 2.60) P = 0.11	
Shen (1999) [20]	Electro-acupuncture N = 41	Prednisolone injection N = 20	Pain relief and range of movement: cured rate based on subjective report	32/41 Cured Cured rate: 78%	7/20 Cured Cured rate: 35%	RR (95% CI) 2.23 (1.20 to 4.14) P = 0.01*	
Zha (2004) [31]	Acupuncture N = 57	Hydrocortisone injection N = 60	Pain relief and range of movement: cured rate based on ADL score	8/57 Cured Cured rate: 14.03%	6/60 Cured Cured rate: 10%	RR (95% CI) 1.40 (0.52 to 3.80) P = 0.5	
Zhang (2007) [32]	Acupuncture N = 36	Meloxicam tablets N = 32	Pain relief and range of movement: cured rate based on subjective report	26/36 Cured Cured rate: 72.2%	15/32 Cured Cured rate: 46.9%	RR (95% CI) 1.54 (1.01 to 2.34) P = 0.04*	

*: P < 0.05 statistically significant difference between two groups. **: Outcomes were measured at the end of last treatment, except for those specifically mentioned.

ADL, Activities of daily living scale; 95% CI, 95% Confidence Interval; DASH score, Disability of the Arm, Shoulder and Hand score; FU, follow-up; RR, Relative Risk; SD, Standard Deviation; MD, Mean Difference; VAS, Visual Analogue Rating Scale.

Ginger-moxibustion was compared to conventional therapy in three trials [22,29,33] (Table 4). Two studies [29,33] used scarring moxibustion, in which the moxa was burned indirectly on the skin, and the area was allowed to heat up to the extent of blister-formation, which would turn into fully formed scar tissue after a period of two to four weeks. The remaining study [22] reported to allow the moxa-cones to heat up the local area until the skin became red and hot to touch, but not to a degree that would cause blister formation. For control treatments, Jin et al. [33] injected vitamin B1 and B12 along with lidocaine and prednisolone into the local site of pain, whereas the two other studies [22,29] used procaine and prednisolone injections.

**Table 4:** Outcomes of randomized controlled trials comparing moxibustion and conventional therapy

**Studies**	**Intervention and number of subjects**		**Outcome measurement**	Outcomes** (Cured no./Total no., Cured rate)		**Treatment effect**
	**Treatment group**	**Control group**		**Treatment group**	**Control group**	**Cured rate: RR (95% CI)**
Jin (2005) [33]	Ginger- moxibustion	Prednisolone injection	Pain relief and range of movement: cured rate based on ADL score	59/80	17/32	1.39 (0.98 to 1.97)
	N = 80	N = 32		73.8%	53.1%	P = 0.07
	
Li (2007) [22]	Ginger- moxibustion	Prednisone injection	Pain relief and grip strength: cured rate based on subjective report	20/25	21/25	0.95 (0.73 to 1.24)
	N = 25	N = 25		80%	84%	P = 0.71
	
Xu (2010) [29]	Ginger- moxibustion	Prednisone injection	Pain relief and grip strength: cured rate based on subjective report	11/23	4/22	2.63 (0.98 to 7.04)
	N = 23	N = 22		47.8%	18.2%	P = 0.05

**: Outcomes were measured at the end of last treatment, except for those specifically mentioned.

ADL, Activities of daily living scale; 95% CI, 95% Confidence Interval; RR, Relative Risk.

Six [23-28] studies compared AMC with acupuncture alone (Table 5). One study [20] compared AMC with electro-acupuncture and local injection of prednisolone using a three-arm design, and its results are presented in both Tables 3 and 5.

**Table 5:** Outcomes of randomized controlled trials comparing Acupuncture and Moxibustion Combined (AMC) with acupuncture

**Studies**	**Intervention and number of subjects**		**Outcome measurement**	Outcomes** (Cured no./Total no., Cured %)		**Treatment effect:**
	**Group 1**	**Group 2**		**Group 1**	**Group 2**	**Cured rate: RR (95% CI)**
Li (1998) [28]	AMC	Acupuncture	Pain relief: cured rate based on subjective report	49/60	15/30	1.63 (1.12 to 2.38)
	N = 60	N = 30		81.7%	50%	P = 0.01*

Liu (2008) [23]	AMC	Acupuncture	Pain relief and range of movement: cured rate based on subjective report	28/37	6/26	3.28 (1.58 to 6.77)
	N = 37	N = 26		76%	23%	P = 0.0013*

Shen (1999) [20]	AMC	Electro-acupuncture	Pain relief and range of movement: cured rate based on subjective report	10/27	32/41	0.47 (0.28 to 0.80)
	N = 27	N = 41		37%	78%	P = 0.005*

Wang (2007) [24]	AMC	Acupuncture	Pain relief and range of movement: cured rate based on subjective report	22/36	9/36	2.44 (1.31 to 4.56)
	N = 36	N = 36		61.1%	25%	P = 0.005*

Wang (2008) [25]	AMC	Acupuncture	Pain relief and range of movement: cured rate based on subjective report	15/32	6/32	2.50 (1.11 to 5.62)
	N = 32	N = 32		46.9%	18.8%	P = 0.03*

Wu (2003) [26]	AMC	Acupuncture	Pain relief and range of movement: cured rate based on subjective report	56/74	13/52	3.03 (1.86 to 4.93)
	N = 74	N = 52		75%	25%	P < 0.0001*

Zhao (2003) [27]	AMC	Acupuncture	Pain relief and range of movement: cured rate based on subjective report	23/25	16/24	1.38 (1.02 to 1.87)
	N = 25	N = 24		92%	66.6%	P = 0.04*

*: P < 0.05 statistically significant difference between two groups. **: Outcomes were measured at the end of last treatment, except for those specifically mentioned.

95% CI, 95% Confidence Interval; AMC, acupuncture and moxibustion combined; RR, Relative Risk.

### Description of acupuncture, moxibustion and acupuncture and moxibustion combined (AMC) regimes

Acupuncture alone was used in ten studies, of which nine [15-19,21,30-32] used manual stimulation and one study [20] employed electro-acupuncture. Moxibustion alone was used in three studies [22,29,33], and AMC was used in seven studies [20,23-28]. The most commonly used acupoints were local tender points (Ashi points) which were used by 14 studies. He Gu (LI 4), Shou San Li (LI 10), and Qu Chi (LI 11) were used in ten studies. Only one study [16] employed an exclusively distal needling approach, in which the ipsilateral Yang Ling Quan (GB 34) acupoint was used. The total number of treatment sessions ranged from one to 36. The frequency of treatments varied from once a day to once every three days and the duration of an entire treatment course lasted from one to 37 days. The number of needles used per session ranged from one to 12 needles. The number of moxa-cones used in the moxibustion and AMC interventions ranged from two to seven cones per acupoint. Of the 19 included studies, 14 reported that De-qi sensation was sought and the duration of each treatment session lasted between one and 30 minutes, with most studies ranging between 20 to 30 minutes.

### Standard in reporting acupuncture treatment

Table 6 presents the standards of reporting acupuncture treatment in the 19 included studies using the revised STRICTA criteria (2010) [11]. None of the included studies reported acupuncture procedure detailed enough to satisfy the STRICTA criteria. Although all the trials reported the methods of acupoint selection, only Fink et al. [15] and Molsberger et al. [16] reported whether needling was bilateral or unilateral. Only three studies [15,16,19] reported the background of the TCM practitioner.

**Table 6:** Appraisal of acupuncture and moxibustion procedure based on the revised SRICTA criteria (2010)

**1st Author (year)**	**Acupuncture rationale**	**Needling details**							**Treatment regime**	**Other components of treatment**		**Practitioner background**	**Control intervention**
		**Points used**	**No. of needles inserted**	**Depths of insertion**	**Responses elicited**	**Needle stimulation**	**Needle retention time**	**Needle type**		**Other components**	**Setting and context**		
Chen (2010) [30]	TCM	Y	NR	Y	Y	Y	Y	NR	Y	NR	NR	NR	Y
Davidson (2001) [18]	TCM	Y	Y	Y	Y	Y	Y	Y	Y	NR	NR	NR	Y
Fink (2002) [15]	TCM	Y*	Y	NR	Y	NR	Y	Y	Y	NR	NR	Y	Y
Grua (1999) [19]	TCM	Y	Y	NR	NR	Y	Y	NR	Y	Y	NR	Y	Y
Irnich (2003) [17]	TCM	Y	Y	NR	NR	Y	Y	NR	Y	NR	NR	NR	Y
Jin (2005) [33]	TCM	Y	NR	NR	Y	Y	NR	NA	Y	NA	NA	NR	Y
Li (1998) [28]	TCM	Y	NR	NR	Y	Y	Y	NA	Y	NA	NA	NR	Y
Li (2007) [22]	TCM	Y	Y	Y	Y	NR	NR	Y	Y	Y	Y	NR	Y
Liu (2008) [23]	TCM	Y	NR	Y	Y	Y	Y	Y	Y	Y	Y	NR	Y
Lin (2011) [21]	TCM	Y	Y	Y	Y	Y	Y	Y	Y	NR	NR	NR	Y
Molsberger (1994) [16]	TCM	Y*	Y	Y	Y	Y	Y	NR	NR	NR	NR	Y	Y
Shen (1999) [20]	TCM	Y	Y	Y	Y	Y	Y	Y	Y	Y	Y	NR	Y
Wang (2007) [24]	TCM	Y	NR	NR	Y	Y	NR	NR	Y	Y	Y	NR	Y
Wang (2008) [25]	TCM	Y	Y	NR	Y	Y	Y	Y	Y	Y	Y	NR	Y
Wu (2003) [26]	TCM	Y	NR	Y	Y	Y	Y	Y	Y	Y	Y	NR	Y
Xu (2010) [29]	TCM	Y	NR	NR	NR	NR	NR	NA	NR	NA	NA	NR	Y
Zha (2004) [31]	TCM	Y	Y	Y	NR	Y	Y	Y	Y	NR	NR	NR	Y
Zhang (2007) [32]	TCM	Y	Y	Y	Y	Y	NR	Y	Y	NR	NR	NR	Y
Zhao (2003) [27]	TCM	Y	NR	Y	Y	Y	NR	Y	Y	Y	Y	NR	Y

NA, not applicable; NR not reported; TCM, acupoint selection based on TCM theory; Y, reported; Y*reported and mentioned if unilateral or bilateral needle placement.

### Quality of included studies and publication bias

#### Assessment by the Cochrane risk of bias tool

Table 7 presents the Cochrane risk of bias assessment. All studies had at least 1 domain rated as high risk of bias, except the study by Fink et al. [15], which had allocation concealment rated as unclear risk of bias. Adequate randomization sequence generation was described in only four studies [15,18,31,32]. Adequate allocation concealment was described in none of the studies. All studies addressed incomplete outcome data adequately and reported all outcome measures. Five studies [20,23,27-29] had not examined between-group imbalance at baseline. The study by Irnich et al. [17] had a significant difference in mean difference at baseline. Only two studies [15,16] had performed power analysis.

**Table 7:** Cochrane risk of bias assessment

**1st Author (year)**	**Random sequence generation**	**Allocation concealment**	**Blinding of participants, personnel, and outcome assessors**	**Complete collection and reporting of outcome data**	**Selective outcome reporting**	**Other sources of bias**
Chen (2010) [30]	Unclear	Unclear	High	Low	Low	Low
Davidson (2001) [18]	Low	Unclear	High	Low	Low	Low
Fink (2002) [15]	Low	Unclear	Low	Low	Low	Low
Grua (1999) [19]	Unclear	Unclear	High	Low	Low	Low
Irnich (2003) [17]	High	High	Low	Low	Low	High
Jin (2005) [33]	Unclear	Unclear	High	Low	Low	Low
Li (1998) [28]	Unclear	Unclear	High	Low	Low	Unclear
Li (2007) [22]	Unclear	Unclear	High	Low	Low	Low
Liu (2008) [23]	Unclear	Unclear	High	Low	Low	Unclear
Lin (2011) [21]	Unclear	Unclear	High	Low	Low	Low
Molsberger (1994) [16]	Unclear	Unclear	High	Low	Low	Low
Shen (1999) [20]	Unclear	Unclear	High	Low	Low	Unclear
Wang (2007) [24]	High	High	High	Low	Low	Low
Wang (2008) [25]	Unclear	Unclear	High	Low	Low	Low
Wu (2003) [26]	Unclear	Unclear	High	Low	Low	Low
Xu (2010) [29]	Unclear	Unclear	High	Low	Low	Unclear
Zha (2004) [31]	Low	Unclear	High	Low	Low	Low
Zhang (2007) [32]	Low	High	High	Low	Low	Low
Zhao (2003) [27]	High	High	High	Low	Low	Unclear

Fourteen of the 19 included studies were conducted in China. As suggested in the study by Wu et al. [14] the adequacy of randomization was investigated by a telephone interview to the authors. We successfully contacted the authors of three studies and found out that the methods of randomization in two studies [24,29] were inadequate, as group assignment was accomplished by alternate assignment. We were also able to reach the author of the Wang et al. (2008) study [25], however at the time of calling the author could not recall any details of the study. The authors of the remaining 11 studies could not be contacted.

Meta-analysis was not performed as all but one study [15] had at least one domain rated as having high risk of bias and measured different outcomes. Examination of publication bias by drawing a funnel plot was not conducted, because comparable studies were less than ten [13].

#### Publication bias

Publication bias was not assessed, as recommended by the Cochrane Handbook for Systematic Review, given the fact that there was not a sufficient amount of studies with adequate similarities in clinical characteristics [13].

### Efficacy assessments

In assessments of efficacy, most studies did not use standard outcome measures such as peak-muscle force, pressure pain threshold, and pain free grip strength to objectively assess outcomes. Instead, they used only subjective outcome measures such as VAS (visual analogue scale) to assess pain, or the DASH (disability of arms, shoulder and hand) questionnaire to assess functional impairment. Objective outcome measurements were only used in three studies [15,17,18].

In addition, all included studies did not differentiate primary and secondary outcome measurements, with the exception of the Fink et al. study [15], which mentioned that all three measurements (maximal strength, pain intensity (verbal rating scale) and DASH) were primary outcomes. Pain intensity in a verbal rating scale was, however, not reported in the outcome parameter table of the published article. Instead, pain was reported in a visual analogue scale (VAS).

Outcomes in all Chinese studies were mainly classified into the following categories: cured, remarkably effective, improved, and ineffective. “Cured” referred to a complete relief of pain and a complete regain of unobstructed range of movement. “Remarkably effective” indicated a general relief of pain and regaining total range of movement, but with occasional reoccurrence of symptoms. “Improved” referred to obvious improvement of pain and range of movement and “ineffective” refers to no improvement. Because the “cured” category appeared to be the only category consistent across all the 14 Chinese studies in assessing treatment efficiency, we analyzed the cured rate in this review. These 14 trials defined the cured rate as the proportion of subjects who achieved complete relief of symptoms related to LEP at the end of the treatment period or during the follow-up. The relative risk (RR) in these studies was calculated for efficacy assessment.

#### Acupuncture vs. Sham-acupuncture

All three studies [15-17] comparing acupuncture against sham acupuncture showed that acupuncture was superior to sham acupuncture for some of the outcome measures, but no study defined the primary outcome measure (Table 2). Molsberger et al. [16] found that subjects in the acupuncture group had a greater pain reduction than those in the sham acupuncture group immediately after treatment as measured by percentage of improvement in pain scale (MD = 40.80, 95% CI: 39.18 to 42.42, P < 0.001) and number of subjects who reported pain relief ≥ 50% (RR = 3.17 CI: 1.53 to 6.52, P < 0.002). Irnich et al. [17] showed that the acupuncture group had a significantly greater reduction in pain and improvement of elbow mobility when compared to the sham acupuncture group immediately after the treatment and at two-week follow-up. Fink et al. [15] reported that reductions in maximal strength, pain intensity, and function of the arm in the acupuncture group were better than those in the sham acupuncture group at 2-week follow up (peak muscle force, measured by a specially designed device for isometric strength, MD = 35.45, 95% CI: 7.42 to 63.48, P = 0.01; pain score, measured by VAS, MD = -4.25, 95% CI: -7.44 to -1.06, P = 0.009; functional impairment, measured by DASH score points, MD = -10.80, 95% CI: -19.26 to -2.34, P = 0.01), but the differences were no longer significant at the 2-month follow-up. Pooled analysis of the three studies was not possible due to incompatible outcome measures.

#### Acupuncture vs. Conventional Therapy

The effects of seven studies comparing acupuncture with conventional therapy are shown in Table 3. Three of the five studies [20,21,30-32] that used dichotomous outcome measurements showed that the acupuncture group had a significantly higher cured rate than conventional therapy, including prednisolone injection [20], triamcinolone acetonide, lidocaine injection [30], and oral administration of meloxicam tablets [32]. The other two studies that compared acupuncture to prednisolone and procaine injection [21], and acupuncture to hydrocortisone acetate injection [31], did not find significant differences between groups. The remaining two studies [18,19] compared acupuncture with pulsated ultrasound, and assessed functional impairment and pain with continuous measures. One of these [19] found significant reduction in functional impairment as measured by the Maigne functional recovery test (MD = -4.80, 95% CI: -7.00 to -2.60, P < 0.001) and significant reduction in VAS pain score (MD = -2.85, 95% CI: -3.73 to -1.97, P < 0.001) in the acupuncture group when compared with the group that received pulsated ultrasound and massage at 6-month follow-up. Due to low methodological quality of most of the included studies, meta-analysis was not performed.

#### Moxibustion vs. Conventional therapy

Three studies compared moxibustion with conventional therapy [22,29,33] and showed no significant difference between the groups (Table 4). Due to low methodological quality of the studies, meta-analysis was not performed.

#### Acupuncture and moxibustion combined (AMC) vs. Acupuncture

Six [23-28] studies comparing AMC with acupuncture alone showed that AMC had a significantly higher cured rate than acupuncture alone. However, a three-arm study by Shen et al. [20] compared AMC with electro-acupuncture showed that the effect of electro-acupuncture was better than AMC (Table 5). Due to low methodological quality of the studies, meta-analysis was not performed.

### Adverse event reporting

Adverse events were only reported in four studies [17,21,29,33]. The studies by Irnich et al. [17] and Grua et al. [19] stated that no adverse event was observed during acupuncture treatment. The studies by Jin et al. [33] and Xu et al. [29] both reported that blister-forming ginger-moxibustion resulted in permanent scar tissue. However, it is unknown if the subjects of the latter two studies have been informed in advance that scarring might result after the course of treatment, in which case the permanent scar tissue might not be considered an adverse event.

## Discussion

The present study reviewed randomized controlled trials on the efficacy of acupuncture and moxibustion treatments for LEP. To our knowledge, this is the first systematic review that included studies in Chinese and studies using moxibustion for LEP. Acupuncture was shown to be more effective than sham acupuncture for treating LEP, up to a period of two months in three randomized subject-blinded clinical trials. However, there is insufficient evidence to conclude that acupuncture or moxibustion is more effective than or as effective as local anesthetic injection, local steroid injection, or non-steroidal anti-inflammatory drugs, due to low methodological quality of these trials and the small sample sizes of individual studies.

Results from reviewed studies that compared manual acupuncture with AMC showed that AMC was more effective than manual acupuncture alone. However, one study found that electro-acupuncture was more effective than AMC. Nevertheless, results from efficacy studies of AMC must be interpreted with caution, as all studies had at least one domain rated as high risk of bias by the Cochrane risk of bias tool. Another potential limitation of this review is that it is not known whether moxibustion alone is equally effective or more effective than acupuncture alone, as we did not find any study comparing acupuncture with moxibustion. We also did not find any study that compared electro-acupuncture with manual acupuncture.

Recently, Vickers et al. [35] conducted a systematic review of 29 RCTs of acupuncture for four chronic pain conditions, including back and neck pain, osteoarthritis, chronic headache, and shoulder pain and performed meta-analysis on individual patient data from 17,922 patients. With the large number of high quality trials, they were able to find a robust effect of acupuncture for each condition, which was superior to both sham and no-acupuncture control groups. Although the present review included many more clinical trials on LEP published in Chinese, we were not able to demonstrate a robust effect of acupuncture due to the fact that all the Chinese trials were of low methodological quality. Nevertheless, compared with previous reviews of acupuncture treatment for LEP, this critical review provides more detailed description of the acupuncture and moxibustion procedures, including acupoint selection, method of stimulation and treatment duration. We found that most studies used local tender points and acupoints of the Large Intestine meridian around the elbow. An interesting finding from our review is that ten out of the 19 included trials involved the use of moxibustion for LEP. However, the rational of selecting acupuncture or moxibustion was not provided in most studies. Since our results suggested that AMC might be more effective than manual acupuncture alone, future clinical trials should be carried out to properly evaluate the effect of moxibustion and AMC, as well as to establish a basis for the selection of treatment methods.

There have been limited studies in human subjects concerning the mechanisms of acupuncture and moxibustion in treatment of LEP. However, there are an increasing number of studies investigating the mechanisms of acupuncture for pain and inflammation, and it is likely that specific acupuncture regimes preferentially involve different mechanisms. Particularly, the use of distal acupoints such as GB 34 may activate diffuse noxious inhibitory control (DNIC) [36] resulting in immediate pain relief, as in the case of the study by Molsberger [16]. Moreover, opioid released from the brain following acupuncture may result in a general analgesic effect [37]. Also, electro-acupuncture can produce non-opioid dependent anti-inflammatory effects via activation of the cholinergic anti-inflammatory pathway [38,39]. At the site of needle insertion acupuncture can also bring upon the release of neuropeptides involved in pain modulation and local vasodilatation, such as calcitonin gene related peptide (CGRP) and substance P [40,41]. It has been suggested that manual acupuncture at the site of needling can produce anti-inflammatory effects via adenosine release [42]. Lastly, acupuncture may increase local blood flow of the target tissue and affect fibroblast migration through myofascial collagen manipulation, both of which are conducive to reverse tendinopathic change in LEP [43].

As for moxibustion, its mechanisms for pain and inflammation are poorly understood, though modulation of inflammatory cytokines resulting from moxibustion has been reported [44]. Release of heat shock protein 70 (Hsp 70) has also been found following moxibustion [45], and Hsp 70 is beneficial for tissue repair [46], which may be advantageous for tendinopathy associated with LEP.

It is apparent from this review that the literature concerning the use of acupuncture and moxibustion for LEP suffered from many drawbacks. All of the included studies, except the study by Fink et al. [15] had at least one domain rated as high risk of bias by the Cochrane risk of bias tool. In particular, the procedure for randomization might not have been conducted appropriately. Of the three Chinese studies that we had succeeded in contacting the authors, none of them were found to be truly randomized. A potential limitation of this review is the fact that we were unable to contact the authors of the remaining 11 studies to determine their adequacy in randomization.

Another major limitation of the studies was blinding, especially in the Chinese studies. Moreover, there was a lack of standardization of outcome measures, no clear statement of primary outcome measure, and much variation in the selection of control treatment, in the duration of treatments, in the duration of observation and in the method of acupuncture or moxibustion treatment. Dropouts and adverse event reporting were absent in most of the trials. Taken together, these limitations may lead to an over estimation of the efficacy and an under estimation of the adverse effects of acupuncture and moxibustion treatment. Therefore, the apparent promising findings from the reviewed studies should be treated with caution.

According to the revised STRICTA criteria, we found some essential details of the acupuncture treatment protocol, such as, number of needles inserted, needle retention time, or needle type, were not reported in about one-third of our included studies. This is not unexpected given the fact that the revised STRICTA criteria were introduced in 2010. The precise description of these components of acupuncture procedure will enable other researchers to replicate the reported treatment protocol accurately and reliably in both research and clinical settings and allow others to appraise the findings critically.

Very few studies in the current review reported adverse events for acupuncture treatment, and those that did, found no adverse event. Taking into consideration the few and mild incidents of adverse events found in previous reports of acupuncture treatment [47-49], it appears that acupuncture is a safe treatment for LEP. However, the safety of moxibustion and AMC remains a concern, as the adverse events have not been properly documented in the reviewed studies and the only two studies [29,33] that mentioned adverse events involved permanent scarring of local skin tissue. Despite this, most adverse events can easily be avoided by standardizing teaching and clinical practices, as a systematic review of Chinese literature from 1956-2010 about adverse events in acupuncture treatment had suggested [50].

One can only postulate the reasons behind the number of low quality studies. For example, the lack of patient acceptance of random allocation and receiving controlled treatment may be one of the major factors, especially in China. This is because acupuncture has been used for thousands of years in China [51] and public awareness of the need for controlled studies may be low. Thus it would be difficult to recruit subjects willing to participate into a truly randomized acupuncture trial. Blinding of the treatment is difficult due to the nature of the acupuncture intervention [52]. Anecdotally, research scholarships, the level of training of research personnel and research culture may all have impacts on the quality of acupuncture trials [14].

There are strengths and methodological limitations of this review. Our updated search covered both English and Chinese databases and was comprehensive enough to identify the current available evidence of acupuncture and/or moxibustion for LEP. However, the breadth of this review would be inevitably limited by a dearth of high quality studies, resulting in some uncertainties regarding the efficacy and safety of acupuncture and moxibustion. Although a number of studies were reviewed, we did not identify any studies comparing AMC with moxibustion alone. Therefore, whether AMC is better than moxibustion alone is still uncertain. Lastly, though we imposed no language restriction in this review, our search did not include databases in other foreign languages such as Korean or Japanese. Thus relevant studies in other foreign languages could not be included in this review.

## Conclusion

Current evidence identified from our review suggested that acupuncture may be effective in the relief of LEP up to a period of six months. Findings from moderate quality studies with subject-blinded and sham-controlled acupuncture intervention groups showed that acupuncture was more effective than sham acupuncture in treatment of LEP. Furthermore, our results also showed that acupuncture in combination with moxibustion was a more effective treatment regime than manual acupuncture alone, but the quality of these studies was low. Taken together, the conclusion of this present review is limited by the fact that most of our included studies had at least one criterion rated as high risk of bias. Therefore, it is premature to draw conclusions on the beneficial effects of acupuncture, moxibustion, or the combination of both for individuals with LEP. Future clinical trials with rigorous design should be conducted to evaluate the efficacy and safety of acupuncture and moxibustion for LEP.

## Competing interest

The authors declare that they have no competing interests. No competing financial or non-financial interest from the funders exist.

## Authors’ contributions

CZ, YST, FCW, SB and SPZ were responsible for the conception and design of this study. HL initially acquired data and performed statistical analysis. WFY and MG performed further data acquisition and extraction, re-analyzed the data and drafted the manuscript. KFC, ZXB and SPZ revised the manuscript. All authors reviewed and approved the final manuscript.

## Pre-publication history

The pre-publication history for this paper can be accessed here:

http://www.biomedcentral.com/1472-6882/14/136/prepub

## References

[B1] Allander E Prevalence, incidence and remission rates of some common rheumatic diseases and syndromes Scand J Rheumatol 1974 3 145 153 10.3109/03009747409097141 4428194

[B2] Peterson M Elmfeldt D Svärdsudd K Treatment practice in chronic epicondylitis: a survey among general practitioners and physiotherapists in Uppsala County Sweden Scand J Prim Health Care 2005 23 4 239 241 10.1080/02813430510031333 16272073

[B3] Smidt N Van der Windt DAWM Assendelft WJJ Devillé WLJM Korthals-de Bos IBC Bouter LM Corticosteroid injections for lateral epicondylitis are superior to physiotherapy and a wait and see policy at short-term follow-up, but inferior at long-term follow-up: results from a randomised controlled trial Lancet 2002 359 657 662 10.1016/S0140-6736(02)07811-X 11879861

[B4] Assendelft W Green S Buchbinder R Struijs P Smidt N Tennis elbow (lateral epicondylitis) Clin Evid 2002 8 1290 1300 12603940

[B5] Karkhanis S Frost A Maffulli N Operative management of tennis elbow: a quantitative review Br Med Bull 2008 88 1 171 188 10.1093/bmb/ldn036 18819957

[B6] Gaujoux-Viala C Dougados M Gossec L Efficacy and safety of steroid injections for shoulder and elbow tendonitis: a meta-analysis of randomised controlled trials Ann Rheum Dis 2009 68 12 1843 1849 10.1136/ard.2008.099572 19054817 PMC2770107

[B7] Hinman RS McCrory P Pirotta M Relf I Crossley KM Reddy P Forbes A Harris A Metcalf BR Kyriakides M Novy K Bennell KM Efficacy of acupuncture for chronic knee pain: protocol for a randomized controlled trial using a Zelen design BMC Complement Altern Med 2012 12 161 10.1186/1472-6882-12-161 22992309 PMC3493360

[B8] Buchbinder R Green SE Struijs P Tennis elbow Clin Evid 2008 2008 1117 PMC2907994 19450309

[B9] Bisset L Paungmali B Vicenzino B Beller E A systematic review and meta-analysis of clinical trials on physical interventions for lateral epicondylagia Br J Sports Med 2005 39 411 422 10.1136/bjsm.2004.016170 15976161 PMC1725258

[B10] Trinh KV Phillips SD Ho E Damsma K Acupuncture for the alleviation of lateral epicondyle pain: a systematic review Rheumatology (Oxford) 2004 43 9 1085 1090 10.1093/rheumatology/keh247 15213328

[B11] MacPherson H Altman DG Hammerschlag R Youping L Taixiang W White A Moher D Revised standards for reporting interventions in clinical trials of acupuncture (STRICTA): extending the CONSORT statement PLoS Med 2010 3 3 140 155 10.1111/j.1756-5391.2010.01086.x 21349059

[B12] Liberati A Altman DG Tetzlaff J Mulrow C Gotzsche PC Ioannidis JP Clarke M Deveraux PJ Kleijnen J Moher D The PRISMA statement for reporting systematic reviews and meta-analyses of studies that evaluate health care interventions: explanation and elaboration BMJ 2009 339 b2700 10.1136/bmj.b2700 19622552 PMC2714672

[B13] Higgins JPT Green S Cochrane handbook for systematic reviews of interventions version 5.1.0 (Updated march 2011) The Cochrane Collaboration 2011

[B14] Wu T Li Y Bian Z Guanjian L Moher D Randomized trials published in some Chinese journals: How many are randomized Trials 2009 10 46 10.1186/1745-6215-10-46 19573242 PMC2716312

[B15] Fink M Wolkenstein E Luennemann M Gutenbrunner C Gehrke A Karst M Chronic epicondylitis: effects of real and sham acupuncture treatment: a randomized controlled patient-and examiner-blinded long-term trial Forsch Komplementarmed Klass Naturheilkd 2002 9 210 215 10.1159/000066030 12232492

[B16] Molsberger A Hille E The analgesic effect of acupuncture in chronic tennis elbow pain Br J Rheumatol 1994 33 1162 1165 10.1093/rheumatology/33.12.1162 8000747

[B17] Irnich D Karg H Behrens N Lang PM Schreiber MA Krauss M Kroeling P Controlled trial on point specificity of acupuncture in the treatment of lateral epicondylitis (tennis elbow) Phys Med Rehab Kuror 2003 13 215 219

[B18] Davidson JH Vandervoort A Lessard L Miller L The effect of acupuncture versus ultrasound on pain level, grip strength and disability in individuals with lateral epicondylitis: a pilot study Physiother Can 2001 53 195 202 211

[B19] Grua D Mattioda A Quirico P Lupi G Allais G Acupuncture in the treatment of lateral epicondylitis: evaluation of the effectiveness and comparison with ultrasound therapy (in Italian) Giornale Italiano di Riflessoterapia ed Agopuntura 1999 11 63 69

[B20] Shen RR 41 cases of acupuncture-moxibustion in comparison to electro-acupuncture in the treatment of lateral epicondylitis (in Chinese) Jilin Chin Med J 1999 4 45

[B21] Lin M 36 Cases of “elbow five needles technique”-acupuncture treatment of lateral epicondylitis (in Chinese) Shandong Chin Med J 2011 30 09 639 640

[B22] Li J Ginger-moxibustion for the treatment of lateral epicondylitis, clinical observations (in Chinese) Clin Acupunct J 2007 23 04 39 40

[B23] Liu J 63 cases of acupuncture-moxibustion in the treatment for lateral epicondylitis (in Chinese) Shandong Med J 2008 48 38 97

[B24] Wang J 72 cases of warm needling acupuncture-moxibustion treatment for lateral epicondylitis (in Chinese) Chin J Tradit Chin Med 2007 19 06 604

[B25] Wang DL 32 cases of warm needle acupuncture-moxibustion in the treatment for lateral epicondylitis (in Chinese) J Clin Anal Med 2008 24 05 36 37

[B26] Wu YZ 74 cases of warm needle acupuncture-moxibustion in the treatment for lateral epicondylitis (in Chinese) Clin Acupunct J 2003 19 04 33 34

[B27] Zhao GM Gao XX Clinical observation of 49 cases of acupuncture-moxibustion treatment of lateral epicondylitis (in Chinese) J Clin Anal Med 2003 19 12 26

[B28] Li AP 60 cases of acupuncture-moxibustion in the treatment for lateral epicondylitis (in Chinese) Shanxi Chin Med J 1998 14 01 29

[B29] Xu LG Ginger moxibustion that leads to blisters treatment of lateral epicondylitis, efficacy observations (in Chinese) China Foreign Med Treat J 2010 22 49

[B30] Chen HS 66 Cases of superficial neddle acupuncture treatment for tennis elbow (in Chinese) Sci Technol Inf J 2010 10 288

[B31] Zha HP Xiong YH Huang WC Superficial needling acupuncture in the treatment of lateral epicondylitis (in Chinese) Chin Acupunct J 2004 24 09 611 612

[B32] Zhang BM Wu YC Acupuncture treatment of tennis elbow, clinical observations China TCM Inf J 2007 14 01 61

[B33] Jin Y 80 cases of suppurative moxibustion treatment of tennis elbow (in Chinese) Zhejiang J Tradit Chin Med 2005 08 362

[B34] Kraushaar BS Nirschl RP Tendinosis of the elbow (tennis elbow). Clinical features and findings of histological, immunohistochemical, and electron microscopy studies J Bone Joint Surg Am 1999 81 2 259 278 10.1302/0301-620X.81B2.9154 10073590

[B35] Vickers AJ Cronin AM Maschino AC Lewith G MacPherson H Foster NE Sherman KJ Witt CM Linde K Acupuncture for chronic pain: individual patient data meta-analysis Arch Intern Med 2012 172 19 1444 1453 10.1001/archinternmed.2012.3654 22965186 PMC3658605

[B36] Le Bars D Dickenson AH Besson JM Diffuse noxious inhibitory controls (DNIC). II. Lack of effect on non-convergent neurones, supraspinal involvement and theoretical implications Pain 1979 6 3 305 327 10.1016/0304-3959(79)90050-2 460936

[B37] Han JS Acupuncture and endorphins Neurosci Lett 2004 361 1–3 258 261 15135942 10.1016/j.neulet.2003.12.019

[B38] Baek YH Choi DY Yang HI Park DS Analgesic effect of electro-acupuncture on inflammatory pain in the rat model of collagen-induced arthritis: mediation by cholinergic and serotonergic receptors Brain Res 2005 1057 1–2 181 185 16139820 10.1016/j.brainres.2005.07.014

[B39] Chung WY Zhang HQ Zhang SP Peripheral muscarinic receptors mediate the anti-inflammatory effects of auricular acupuncture Chin Med 2011 6 1 3 10.1186/1749-8546-6-3 21251313 PMC3033863

[B40] Carlsson CP Sundler F Wallengren J Cutaneous innervation before and after one treatment period of acupuncture Br J Dermatol 2006 155 5 970 976 10.1111/j.1365-2133.2006.07450.x 17034527

[B41] Kashiba H Ueda Y Kashiba H Ueda Y Acupuncture to the skin induces release of substance P and calcitonin gene-related peptide from peripheral terminals of primary sensory neurons in the rat Am J Chin Med 1991 19 189 197 10.1142/S0192415X91000260 1722639

[B42] Goldman N Chen M Fujita T Xu Q Peng W Liu W Jensen TK Pei Y Wang F Han X Chen JF Schnermann J Takano T Bekar L Tieu K Nedergaard M Adenosine A1 receptors mediate local anti-nociceptive effects of acupuncture Nat Neurosci 2010 13 7 883 888 10.1038/nn.2562 20512135 PMC3467968

[B43] Neal BS Longbottom J Is there a role for acupuncture in the treatment of tendinopathy Acupunct Med 2012 30 40 346 349 22918022 10.1136/acupmed-2012-010208

[B44] Kogure M Mimura N Ikemoto H Ishikawa S Nakanishi-Ueda T Sunagawa M Hisamitsu T Moxibustion at mingmen reduces inflammation and decreases IL-6 in a collagen-induced arthritis mouse model J Acupunct Meridian Stud 2012 5 1 29 33 10.1016/j.jams.2011.11.004 22309905

[B45] Kobayashi K Kobayashi K Induction of heat-shock protein (hsp) by moxibustion Am J Chin Med 1995 23 3–4 327 330 8571930 10.1142/S0192415X95000390

[B46] Kovalchin JT Wang R Wagh MS Azoulay J Sanders M Chandarwarkar RY In vivo delivery of heat shock protein 70 accelerates wound healing by up-regulating macrophage-mediated phagocytosis Wound Repair Regen 2006 14 2 129 137 10.1111/j.1743-6109.2006.00102.x 16630101

[B47] Witt CM Pach D Reinhold T Wruck K Brinkhaus B Mank S Willich SN Treatment of the adverse effects from acupuncture and their economic impact: a prospective study in 73,406 patients with low back or neck pain Eur J Pain 2011 15 2 193 197 10.1016/j.ejpain.2010.06.008 20609604

[B48] Lao L Hamilton GR Fu J Berman BM Is acupuncture safe? A systematic review of case reports Altern Ther Health Med 2003 9 1 72 83 12564354

[B49] Witt CM Pach D Brinkhaus B Wruck K Tag B Mank S Willinch SN Safety of acupuncture: results of a prospective observational study with 230 patients and introduction of a medical information and consent form Forsch Komplementmed 2009 16 2 91 97 10.1159/000209315 19420954

[B50] He W Zhao X Li Y Xi Q Guo Y Adverse events following acupuncture: a systematic review of the Chinese literature for the years 1956-2010 J Altern Complement Med 2012 18 10 892 901 10.1089/acm.2011.0825 22967282

[B51] Yuan HW Ma LX Qi DD Zhang P Li CH Zhu J The historical development of deqi concept from classics of traditional Chinese medicine to modern research: exploitation of the connotation of deqi in Chinese medicine Evid Based Complement Alternat Med 2013 2013 639302 24302968 10.1155/2013/639302 PMC3835614

[B52] Finniss DG Kaptchuk TJ Miller F Benedetti F Biological, clinical, and ethical advances of placebo effects Lancet 2010 375 9715 686 695 10.1016/S0140-6736(09)61706-2 20171404 PMC2832199

